# FLAG Immunoprecipitation-Based Mapping of the In Vivo Assembled Spliceosomal C* Complex

**DOI:** 10.3390/ijms26209914

**Published:** 2025-10-12

**Authors:** Sweta Kumari, Kusum K. Singh

**Affiliations:** Department of Biosciences and Bioengineering, Indian Institute of Technology Guwahati, Guwahati 781039, Assam, India; sweta176106105@iitg.ac.in

**Keywords:** spliceosomal complexes, BioID, MINX, FLAG, RNP immunoprecipitation

## Abstract

Pre-mRNA splicing is catalyzed by the ribonucleoprotein (RNP) complex known as the spliceosome. The spliceosomes are dynamic and undergo constant rearrangement, leading to the formation of the different spliceosomal complexes A, B, B^act^, C, C*, and P. Isolation of the spliceosomal complex at a specific intermediate stage requires a means to enrich it. This study describes a strategy for studying intermediate spliceosomal complexes by combining BioID with splicing assays. The MINX splicing substrate with a mutation at the 3′ splice site was utilized to arrest and capture the spliceosomal C* complex before the second catalytic step of splicing. The splicing substrate also contains binding sites for the MS2 coat protein, which facilitates the pull-down of assembled complex by FLAG-MS2-tagged RNP immunoprecipitation and determines the captured proximal proteins by mass spectrometry.

## 1. Introduction

Pre-mRNA splicing is catalyzed by the spliceosome, a highly dynamic ribonucleoprotein complex composed of uridine-rich snRNPs (U1, U2, U4, U5, U6) and numerous auxiliary proteins [1,2,3]. Spliceosome assembly proceeds through a series of intermediates, E, A, pre-B, B^act^, B*, C, C*, and P complexes, each defined by specific snRNP composition and conformational rearrangements. Catalysis occurs in two sequential steps: in the B* complex, the branchpoint adenosine attacks the 5′ splice site, generating the C complex with a cleaved 5′ exon and intron lariat-3′ exon; subsequently, in the C* complex, exon ligation is completed to yield mature RNA [1,4,5].

A defining feature of late-stage spliceosomes is the deposition of the exon junction complex (EJC) near exon–exon junctions of spliced mRNAs [6,7,8]. The EJC core comprises eIF4A3, MAGOH, and RBM8A/Y14 [9,10,11]. Structural studies have revealed that stable EJCs are assembled within the spliceosomal C* complex [12,13]. In addition to the core, peripheral factors, such as SAP18, have been identified by mass spectrometry as EJC-associated proteins [10]. However, the mechanism by which SAP18 is recruited to the EJC, and whether it remains associated during catalytically active complexes, has remained unclear.

Our recent work using BioID-based proximity labeling demonstrated that SAP18 is recruited as early as the prespliceosomal A complex stage [14], earlier than previously expected. This raises the unresolved question of whether SAP18 persists through later stages of spliceosome assembly, particularly within the catalytically active C and C* complexes, and how it may interact with EJC core components during exon ligation.

To investigate whether SAP18 remains associated with the EJC at the spliceosomal C* complex, we generated a BirA*-SAP18 fusion protein by tagging SAP18 with myc-BioID. This approach enables the in vivo identification of proximal proteins, including those involved in weak or transient interactions, making it an ideal approach for elucidating SAP18′s role during dynamic spliceosomal rearrangements. In combination, we employed in vivo splicing assays utilizing the mutant MINX-GG-MS2 reporter plasmid, which stalls at the C* complex stage. By combining BioID with in vivo splicing assays and subsequent FLAG immunoprecipitation to isolate the assembled complex, we identified novel SAP18-associated interactions within the C* complex.

## 2. Results

### 2.1. Design of the MS2-Based In Vivo Splicing Reporter System

**Figure 1:**
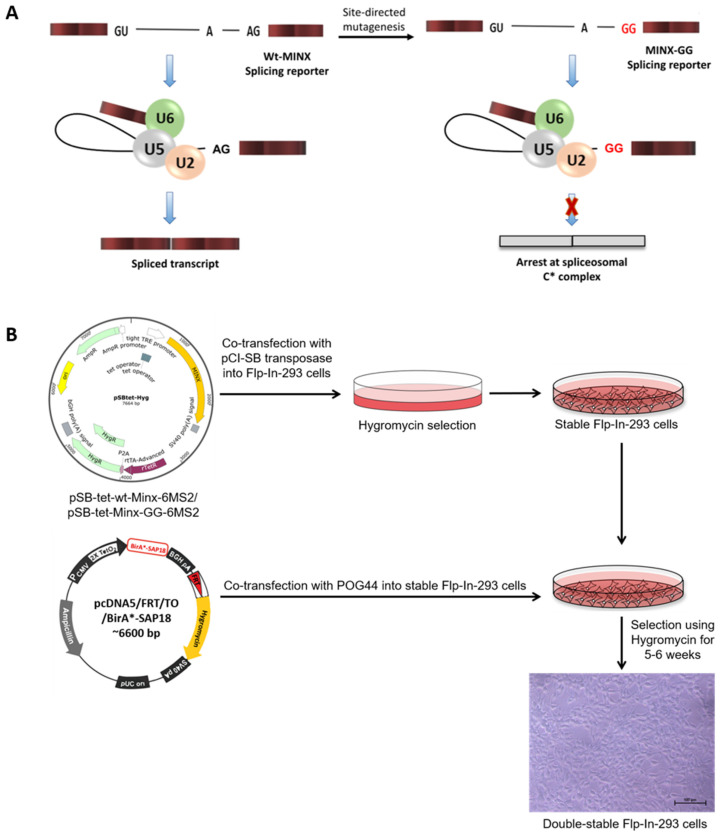
Design of the MS2-based in vivo splicing reporter system. (**A**) Design of MINX splicing reporters for the in vivo splicing assays. Normal MINX pre-mRNA with GU appears at the 5′ splice site and AG at the 3′splice site, whereas mutant MINX pre-mRNA with GU appears at the 5′ splice-site and the mutant GG at the 3′ splice site. (**B**) Schematic diagram showing the generation of double-stable cells expressing both the MINX splicing reporter and BirA*-SAP18 fusion. Scale bar: 100 µm.

Splicing assays are generally performed on a model pre-mRNA; therefore, we employed the MINX pre-mRNA containing a short intron flanked by two exons. Two versions of the MINX construct were utilized: wild-type MINX pre-mRNA having an intact 3′ splice site and a mutant version harboring a GG substitution at the 3′ splice site (Figure 1A). The GG mutation disrupts the 3′ splice site, thereby preventing the completion of splicing after intron lariat formation and allowing for the accumulation and study of intermediate spliceosomal complexes, specifically the C* complex. To enable the purification of these stalled complexes, the MINX pre-mRNAs were engineered to include an affinity tag composed of six MS2 RNA hairpins (6xMS2), which are recognized by the bacteriophage MS2 coat protein (MS2cp). Using the Sleeping Beauty (SB) transposon system, we stably integrated the plasmids pSB-wt-MINX-6MS2 and pSB-MINX-GG-6MS2 into the genome of Flp-In-293 cells [15]. Stable integrants were selected using hygromycin resistance for 5–6 weeks (Figure 1B). Additionally, to investigate the role of SAP18 in spliceosome dynamics, we integrated a myc-BirA*-SAP18 fusion construct into the genome of the Flp-In-293 cells using the Flp recombinase-mediated recombination system. Stable cell lines expressing BirA*-SAP18 were selected using hygromycin over 5–6 weeks (Figure 1B).

**Figure 2:**
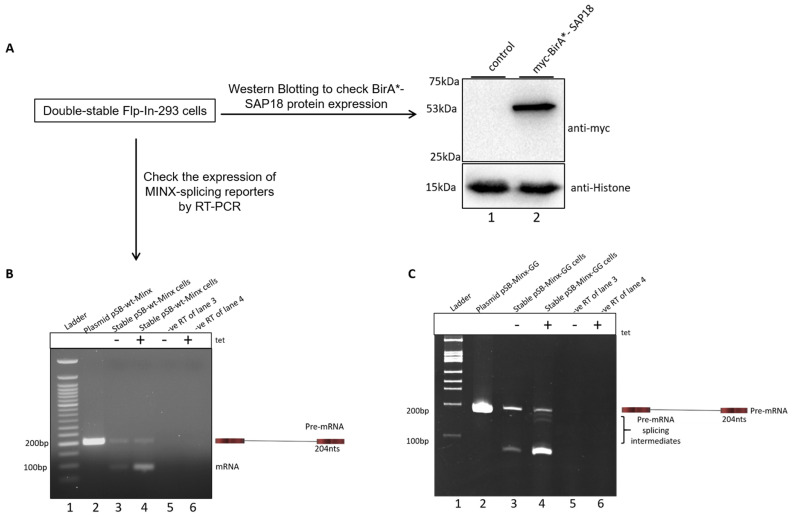
Double stable cells expressing the BirA*-SAP18 and MINX splicing reporters. (**A**) Western blotting analysis confirmed the expression of BirA*-SAP18 in the membrane probed with α-myc antibody (Lane 2). Here, untransfected cells in lane 1 serve as a control. (**B**) Agarose gel electrophoresis showing the RT-PCR of the wt-MINX splicing reporter. Lane 2 contains plasmid DNA used for double-stable cell generation. Lanes 3 and 4 show the bands of unspliced and spliced transcripts. Compared to lane 3, lane 4 shows an enhanced expression of spliced transcripts in the presence of tetracycline (tet). Lanes 5 and 6 are the negative RT controls of lanes 3 and 4, suggesting no genomic DNA contamination in the synthesized cDNA. (**C**) Gel electrophoresis showing the RT-PCR of the MINX-GG splicing reporter. Lane 2 contains plasmid DNA used for double-stable cell generation. Lanes 3 and 4 show the bands of unspliced (204 nts) and pre-mRNA splicing intermediates, including intron lariat-3′exon (145 nts) and cleaved 5′-exon (59 nts). Lanes 5 and 6 are the negative RT controls of lanes 3 and 4, suggesting no genomic DNA contamination in the synthesized cDNA.

To confirm the successful integration and inducible expression of both the splicing reporters and BirA*-SAP18, we treated the double-stable cell lines with 2 μg/mL tetracycline. Expression of the BirA*-SAP18 fusion protein was verified by Western blotting (Figure 2A). Subsequently, RT-PCR analysis was performed using specific primers to detect MINX transcript expression (Figure 2B,C). Agarose gel electrophoresis of RT-PCR products from the wt-MINX reporter showed a 204 nts unspliced pre-mRNA band and a 99 nts spliced transcript band (Figure 2B). The second lane contains the 204 nts product amplified from plasmid DNA, serving as a positive control. Enhanced spliced transcript levels were observed in tetracycline-induced samples (lane 4), compared to uninduced controls (lane 3). Expression from the MINX-GG reporter was also assessed (Figure 2C). Gel electrophoresis of the RT-PCR products revealed three distinct bands. The full-length band corresponded to the unspliced transcript, with an expected size of 204 nucleotides. In addition, two smaller bands were observed, representing splicing intermediates: an intron lariat-3′ exon fragment of 145 nucleotides and a cleaved 5′ exon fragment of 59 nucleotides. The second lane contains plasmid-derived positive control. Lanes 5 and 6 in both panels (Figure 2B,C) represent negative RT controls for lanes 3 and 4, respectively, confirming the absence of genomic DNA contamination in the cDNA samples. Collectively, these results confirm the successful generation of double-stable Flp-In-293 cells that inducibly express both the MINX splicing reporters and BioID-tagged SAP18, enabling the investigation of spliceosomal dynamics and protein interactions during splicing.

### 2.2. Mapping the Spliceosomal Complex Assembled on MINX Reporters by RNP IP

**Figure 3:**
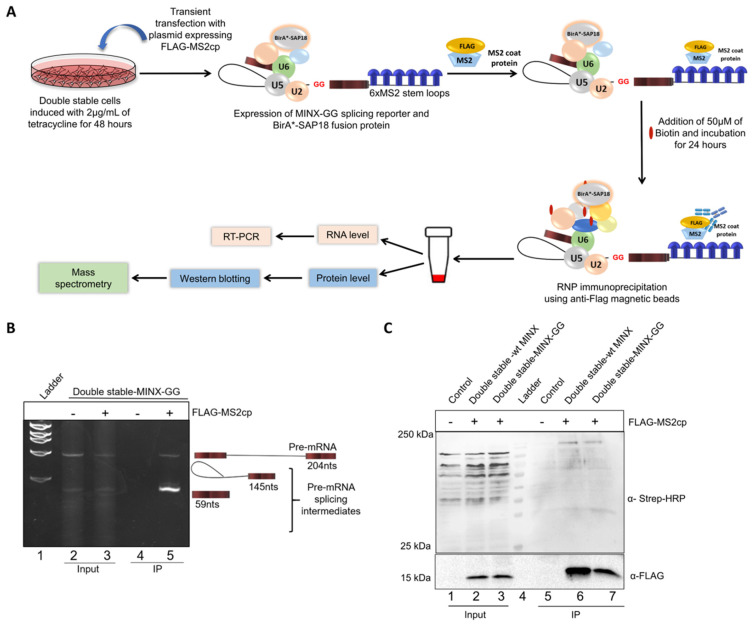
Validation of splicing-specific interactome captured by RNP IP. (**A**) Workflow for FLAG immunoprecipitation-based mapping of the in vivo assembled spliceosomal C* complex. (**B**) Gel electrophoresis showing the RT-PCR of the MINX-GG splicing reporter from RNP IP samples. Lanes 2 and 3 show the bands of pre-mRNA and splicing intermediates of MINX-GG in the input samples. Compared to input samples in lane 3, lane 5 shows the enrichment of the splicing intermediates in the IP sample. Lanes 2 and 4 serve as a negative control because, in the absence of FLAG-MS2cp, no pull-down of the MINX-GG reporter takes place. (**C**) Western blotting analysis of the RNP complex assembled on the MINX splicing reporters. The 15 kDa bands of FLAG-MS2cp in lanes 6 and 7 of IP confirm the MS2-tagged affinity capture of the interactome. IP samples in lanes 6 and 7 confirm the pull-down of biotinylated proteins compared to the biotinylated proteins in all input lanes.

As previously described, the double-stable Flp-In-293 cell lines inducibly express both the BirA*-SAP18 fusion protein and the MINX splicing reporter upon tetracycline treatment. The MINX reporter transcript includes a 3′ MS2-binding site (MS2bs), enabling the affinity purification of RNA-protein complexes through MS2-mediated capture. MS2bs is a highly characterized 19-nucleotide viral RNA sequence that forms a stem–loop structure specifically recognized with high affinity by the MS2 bacteriophage coat protein (MS2cp) (Figure 3A). To facilitate RNP complex capture, double-stable cells were transiently transfected with the pCI-FLAG-MS2cp expression vector, which encodes FLAG-tagged MS2cp with an affinity for 6xMS2bs present in the MINX transcript. Upon expression, the MINX reporter undergoes in vivo splicing by the endogenous spliceosome machinery in the presence of BirA*-SAP18. Proteins in close proximity to SAP18 during splicing are biotinylated via the BirA* enzyme and can be captured as part of the RNP complex through FLAG-based immunoprecipitation using anti-FLAG M2 magnetic beads. The eluted complexes were subsequently analyzed at both RNA and protein levels prior to mass spectrometry (Figure 3A).

At the RNA level, total RNA was extracted from the immunoprecipitated (IP) complexes and reverse-transcribed into cDNA. The presence of MINX pre-mRNA splicing intermediates was evaluated via RT-PCR. Compared to the input lane 3, IP lane 5 in Figure 3B shows a strong enrichment of splicing intermediates, along with residual unspliced pre-mRNA, indicating the successful pull-down of post-lariat splicing intermediates associated with the C* complex. These data suggest that while the majority of captured complexes represent the C* stage, a minor fraction may include earlier spliceosomal intermediates (A to C complexes). In lane 2, one can observe an input where the FLAG-MS2cp protein is not expressed; hence, no pull-down of MINX-GG transcripts is observed in the corresponding IP lane 4, serving as the negative control (Figure 3B).

At the protein level, immunoprecipitated samples were analyzed by Western blotting (Figure 3C). Probing with an anti-FLAG antibody revealed a 15 kDa band corresponding to FLAG-MS2cp in lanes 6 and 7, confirming the successful affinity capture of the MS2-tagged RNP complexes. The same samples also showed strong streptavidin–HRP signals, indicating the presence of biotinylated proteins (lanes 6–7, Figure 3C). In contrast, the control IP sample (lane 5) lacked both FLAG-MS2cp and biotin signals, validating the specificity of the IP and serving as a negative control. Following the confirmation of a successful pull-down, IP samples from lane 7 (test) and lane 5 (control) were subjected to mass spectrometry analysis. Enriched proteins exhibiting biotinylation were identified and selected for further characterization as putative interactors of SAP18 within the spliceosomal C* complex.

### 2.3. Identification of the Spliceosomal Proteins Enriched on the MINX-GG Splicing Reporter by Mass Spectrometry

**Figure 4:**
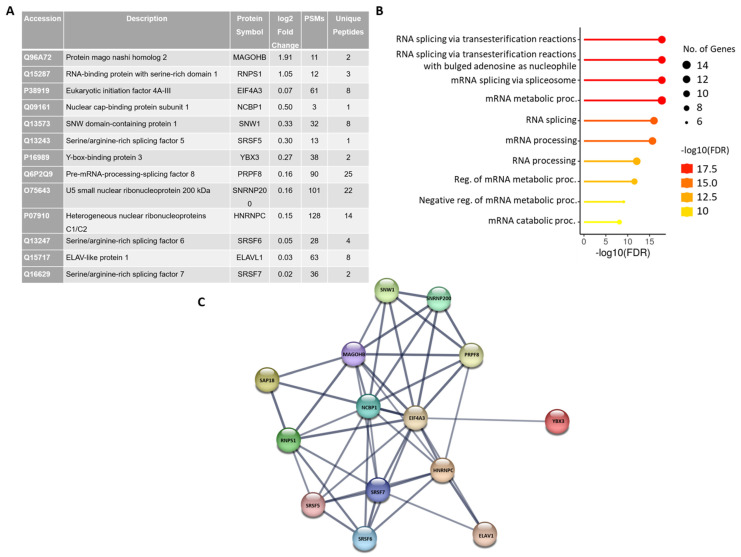
Mapping of enriched spliceosomal proteins. (**A**) List of the spliceosomal proteins enriched on the MINX-GG splicing reporter and identified by MS. (**B**) Classification of enriched proteins by their gene ontology biological process (GOBP) terms: RNA splicing via transesterification reactions, mRNA splicing via spliceosome, RNA splicing, and others. A lollipop plot was generated using ShinyGO 0.81. (**C**) STRING interaction network of enriched proteins with a high interaction score (confidence: 0.700). Each node represents individual proteins. Lines between nodes represent direct and indirect associations of proteins. The strength of association is indicated by the thickness of the line. This network was generated by STRING: functional protein association networks.

Mass spectrometry analysis of RNA–protein complexes assembled on the MINX-GG splicing reporter revealed a significant enrichment of spliceosomal C/C* complex components (Supplementary Table S1, Figure 4A). Notably, the BioID-based pull-down of BirA*-SAP18 identified multiple late-stage spliceosomal proteins, including U5 snRNP-associated factors, such as PRPF8 and SNRNP200, as well as core constituents of the EJC, including MAGOHB, RNPS1, and eIF4A3. The co-enrichment of these factors suggests that SAP18 resides in close proximity to the C* complex and may contribute to the recruitment or stabilization of the EJC during the late stages of spliceosome assembly (Supplementary Figures S1 and S2). This finding is consistent with cryo-EM structural studies that localize the EJC near the catalytic core of the spliceosome [13], supporting a model in which SAP18 facilitates EJC deposition onto spliced transcripts.

Gene Ontology Biological Process (GOBP) analysis of the enriched protein set showed a significant overrepresentation of splicing-associated terms, including RNA splicing via transesterification reactions (GO:0000375), mRNA splicing via spliceosome (GO:0000398), and mRNA metabolic processing (GO:0016071) (Figure 4B), further corroborating the spliceosomal context of the SAP18 interactome.

Protein–protein interaction analysis using the STRING database highlighted a coherent interaction network comprising known SAP18 partners and spliceosomal components (Figure 4C). Among the enriched proteins were EJC constituents (eIF4A3, MAGOHB, and RNPS1), core U5 snRNP proteins (PRPF8, SNRNP200), SR proteins (SRSF5, SRSF6, and SRSF7), the cap-binding complex protein NCBP1, and PRP19 complex-associated factor SNW1. Additionally, other RNA-binding and splicing factors, such as HNRNPC, YBX3, and ELAVL1, were also identified. STRING interaction scores indicate particularly strong associations between SAP18 and EJC-associated proteins RNPS1 and MAGOHB within the captured C* complex, suggesting a potential role for SAP18 in mediating EJC–spliceosome coupling during the late splicing stage.

## 3. Discussion

Deciphering the interactome of the spliceosomal complex is important for understanding the compositional and structural dynamics that govern pre-mRNA splicing. While extensive structural and proteomic studies have illuminated various aspects of spliceosome architecture and function, several key questions remain unresolved, particularly regarding the recruitment and integration of peripheral factors associated with the EJC. Previous reports have demonstrated that core EJC components are recruited by spliceosomal proteins such as CWC22 and CWC27 [7,16,17,18,19,20]. Furthermore, cryo-electron microscopy studies of the spliceosomal C* complex have revealed the positioning of the EJC within the catalytic core of the spliceosome and have uncovered novel interactions involving CWC22, PRP8, EFTUD2, and SLU7 [13,21]. However, mechanisms governing the recruitment of peripheral EJC-associated proteins, such as SAP18, remain poorly understood. In this study, we employed a dual approach that combines BioID-based proximity labeling and MS2-tagged RNA affinity purification to identify proteins associated with SAP18 during spliceosome assembly. The stable expression of a BirA*-SAP18 fusion protein allowed us to capture transient and weak interactions that are often lost in conventional immunoprecipitation (IP) protocols. Simultaneously, the use of MS2-tagged MINX-GG reporter RNA enabled the selective isolation of RNP complexes assembled in vivo. The high-affinity interaction between the MS2 coat protein (MS2cp) and its cognate RNA stem–loop (K_d_ values < 1 nM) facilitated the efficient pull-down of the spliceosomal complex, thus enriching the proteins in close proximity to SAP18 within the C* complex.

Mass spectrometry analysis of the purified RNP complexes revealed a strong enrichment of EJC components, particularly MAGOHB and RNPS1, in association with SAP18. These findings suggest that SAP18 is positioned in close proximity to the EJC core within the C* complex and may contribute to the stabilization or functional integration of the EJC during late-stage spliceosome assembly. Moreover, SAP18’s detection in both the early (A) and late (C*) spliceosomal complexes implies a potential adaptor role, guiding the recruitment of EJC components across splicing intermediates. In addition to EJC constituents, we identified a broad array of spliceosomal proteins, including U5 snRNP factors (PRPF8, SNRNP200), cap-binding protein NCBP1, PRP19 complex-associated SNW1, and splicing regulators, such as HNRNPC, YBX3, ELAVL1, and SR proteins (SRSF5, SRSF6, and SRSF7). The presence of these factors supports the notion that peripheral EJC-associated proteins may be incorporated at multiple stages of the splicing cycle, rather than as a single discrete recruitment event. This multi-step assembly may be critical for ensuring proper EJC deposition and downstream mRNA surveillance or export processes.

While our study advances our understanding of SAP18’s interactions within the spliceosome and its potential role in EJC recruitment, several questions remain. Future studies will benefit from complementary approaches to clarify the mechanistic role of SAP18 in spliceosomal and EJC assemblies. In vitro splicing assays using recombinant SAP18 together with defined EJC core proteins could directly test its role in EJC deposition and exon ligation. In fact, Schwerk et al. demonstrated that SAP18 possesses splicing-enhancing activity, supporting the feasibility of such reconstitution experiments [22]. Similarly, loss-of-function strategies, such as RNA interference or CRISPR-based gene editing, would be valuable for validating the function of SAP18 in vivo, especially in relation to spliceosome dynamics. In this context, Boehm et al. showed that the depletion of SAP18 impedes splicing, reinforcing its regulatory importance [23]. Finally, high-resolution structural methods, including cryo-EM and cross-linking mass spectrometry focused on SAP18-EJC interactions, could provide insights into its precise spatial arrangement within late-stage spliceosomal complexes. Together, these complementary strategies would establish a more complete mechanistic framework for understanding how SAP18 contributes to splicing regulation.

## 4. Materials and Methods

### 4.1. Mammalian Cell Culture

Flp-In^TM^-293 cell lines (R750-07, Thermo Fisher Scientific, Waltham, MA, USA) were cultured in Dulbecco Modified Eagle Medium (DMEM) (AT007F, HiMedia, Kennett Square, PA, USA) supplemented with 10% (*v*/*v*) fetal bovine serum (FBS) (RM10432, HiMedia, Kennett Square, PA, USA) and 1% penicillin–streptomycin solution (A014, HiMedia, Kennett Square, PA, USA) at 37 °C in humidified condition with 5% CO_2_ supply.

### 4.2. Plasmids

The MINX splicing reporter is an adenovirus major late pre-mRNA derivative (MINX) containing a short intronic sequence flanked by two exons. The MINX-6MS2 fragment is cloned in the pSBtet-Hyg (Addgene plasmid #60508; Watertown, MA, USA) expression plasmid. The plasmid vector, pSB-MINX-GG-6MS2, was generated by site-directed mutagenesis using pSB-MINX-6MS2 as a template for point mutation at the 3′ splice site from AG to GG. The mutated vector was confirmed by Sanger sequencing. The plasmid vector pCI-FLAG-MS2cp expressing the MS2 coat protein was employed as it has an affinity for MS2 stem loops required for RNP immunoprecipitation.

### 4.3. Generation of Double-Stable Flp-In^TM^-293 Cells Expressing BioID Fusion Proteins and Splicing Reporter

Flp-In^TM^-293 cells were transfected for the integration of the splicing reporter by using the Sleeping Beauty (SB) transposon system [15]. Approximately 5 × 10^5^ stable Flp-In-293 cells (expressing BirA*-SAP18) were seeded in 6-well plates and co-transfected with 2.0 μg splicing reporter constructs, pSBtet-Hyg-wt-MINX-6MS2/pSBtet-Hyg-MINX-GG-6MS2, and a 1.5 μg SB transposase vector. Forty-eight hours after transfection, the cells were transferred to 60 mm dishes and selected with DMEM supplemented with 200 μg/mL hygromycin (HiMedia). Selection in a hygromycin-containing medium was continued for approximately 5–6 weeks, and resistant colonies were picked and expanded. These stable cells were further co-transfected with a plasmid vector, pcDNA5/FRT/TO/myc-BirA*-SAP18 and 2.0 μg pOG44 (the expression vector for the FLP recombinase), for the Flp recombinase-mediated integration of BirA*-SAP18 inside the genome of Flp-In-293 cells. In these transfected cells, hygromycin selection was continued for approximately 5–6 weeks until single hygromycin-resistant colonies became visible in the cell culture dish. Single colonies were picked and expanded, leading to the generation of double-stable cell lines. In 2 μg/mL tetracycline, both BioID fusion proteins and the splicing reporter were constitutively expressed.

### 4.4. RNA Isolation and cDNA Synthesis

Total cellular RNA was extracted with TRIzol reagent (Invitrogen, Carlsbad, CA, USA) and chloroform separation. In order to remove genomic DNA contamination from isolated RNA, total RNA was incubated with DNase I (10 U/µL, Promega, Madison, WI, USA). A total of 1 µL of DNase was added to 2 µg of total RNA and incubated at 37 °C for 30 min. Then, DNase was inactivated with 1 µL of DNase stop solution and incubated at 65 °C for 10 min.

DNase-treated total RNA was used for cDNA synthesis by the high-capacity cDNA reverse transcription kit (Applied Biosystems, Waltham, MA, USA). A total of 2 µg of total RNA was reverse-transcribed to cDNA with oligo dT and recombinant Moloney murine leukemia virus reverse transcriptase.

### 4.5. Reverse Transcriptase PCR (RT-PCR)

MINX splicing reporters were amplified (98 °C 10 s, 68 °C 15 s, 72 °C 20 s, 30 cycles) using the Taq 2× master mix (Takara, Kusatsu, Shiga, Japan). cDNA prepared in Section 4.4 was used as a template. Forward and reverse primers used for the amplification were specific to both ends of the splicing reporter. The unspliced and spliced products were separated on 1.5% (*w*/*v*) agarose gel. The gel was stained with ethidium bromide (EtBr) and visualized in the ChemiDoc XRS+ gel imaging system (Bio-Rad, Hercules, CA, USA).

### 4.6. RNP Immunoprecipitation (RNP IP)

For MS2-based RNP immunoprecipitation, double-stable Flp-In-293 cells expressing the MINX splicing reporter and BioID fusion proteins were used to capture the spliceosomal complex. Approximately 2 × 10^6^ cells were seeded in 10 cm dishes. Twenty-four hours post-seeding, cells were transfected with 5 µg of pCI-FLAG-MS2cp and 1.0 µg pCI-mVenus as a transfection control using PEI transfection reagent. Transfected cells were cultured in the presence of 2 μg/mL tetracycline (HiMedia) for 48 h. Next, cells were supplemented with 50 μM biotin (HiMedia) and were harvested 24 h later. To this end, the cells were washed thrice with 2–3 mL 1 × DPBS, scraped from the dish in 1000 µL of RIP buffer (25 mM Tris pH 8.0, 150 mM NaCl, 1 mM EDTA, 1% deoxycholate, 5% Glycerol, 1X CPI (complete protease inhibitor cocktail (Sigma-Aldrich) plus 2.5 µL of RNasin (40 U/µL) (Promega, N2611) and transferred to a 1.5 mL microcentrifuge tube. Cells were lysed by sonication (15 cycles at 25% amplitude; 1 s ON, 4 s OFF) using an Ultrasonic Homogenizer (Fisher Scientific)). The homogenate was centrifuged at 13,000 rpm for 10 min at 4 °C and transferred to a fresh 1.5 mL microcentrifuge tube. Then, 10% lysate was set aside as an input for RNA isolation and Western blotting. Subsequently, 90% lysate was incubated with 20 µL anti-FLAG-M2 magnetic beads (Sigma-Aldrich, Saint Louis, MO, USA) for 6 h in Rotospin (Tarsons, Kolkata, West Bengal, India) at 4 °C. After incubation, the beads were washed 3 times with 600 µL of RIP buffer using a magnetic stand (Promega). After the last wash, the beads were divided into two fractions to elute the RNP complex attached to them. One fraction was incubated at 95 °C for 10 min with 5 × Laemmli SDS sample buffer for protein analysis. The second fraction was dissolved in Trizol (Invitrogen, 15596018) for RNA isolation and further confirmation of the MINX reporter by RT-PCR.

### 4.7. Mass Spectrometric Analysis and Data Processing

The sample was used for digestion, reduced with 5 mM of TCEP, further alkylated with 50 mM of iodoacetamide, and then digested with Trypsin (1:50, Trypsin/lysate ratio) for 16 h at 37 °C. The digest was cleaned using a C18 silica cartridge to remove the salt and dried using a speed vac. The dried pellet was resuspended in buffer A (2% acetonitrile, 0.1% formic acid).

The experiment was performed on an Easy-nlc-1000 system coupled with an Orbitrap Exploris mass spectrometer (Thermo Fisher Scientific, Waltham, MA, USA). A total of 1 μg of peptide sample was loaded on C18 column, 13 cm, 3.0 μm Acclaim PepMap (Thermo Fisher Scientific, Waltham, MA, USA), and separated with a 0–40% gradient of buffer B (80% acetonitrile, 0.1% formic acid) at a flow rate of 500 nL/min. Moreover, it was injected for MS analysis. LC gradients were run for 110 min. MS1 spectra were acquired in Orbitrap (Max IT = 60 ms, AGQ target = 300%; RF Lens = 70%; R = 60 K, mass range = 375–1500; Profile data). Dynamic exclusion was employed for 30 s, excluding all charge states for a given precursor. MS2 spectra were collected for the top 20 peptides. MS2 values were Max IT = 60 ms, R = 15 K, AGC target 100%. The sample was processed, and the generated RAW files were analyzed using Proteome Discoverer (v2.5) against the Uniprot database. For the dual Sequest and Amanda search, the precursor and fragment mass tolerances were set at 10 ppm and 0.02 Da, respectively. The protease used to generate peptides, i.e., enzyme specificity, was set for Trypsin/P (cleavage at the C terminus of “K/R: unless followed by “P”). Carbamidomethyl on cysteine as a fixed modification and the oxidation of methionine, N-terminal acetylation, and lysine for biotinylation were considered as variable modifications for the database search. Both peptide spectrum match and protein false discovery rate were set to 0.01 FDR.

## Data Availability

The data presented in this study are available on request from the corresponding author.

## Supplementary Materials

The following supporting information can be downloaded at: https://www.mdpi.com/article/10.3390/ijms26209914/s1.
