# Supplementary material for: FLAG Immunoprecipitation-Based Mapping of the In Vivo Assembled Spliceosomal C* Complex
Source: Int J Mol Sci. 2025 Oct 12;26(20):9914. doi: 10.3390/ijms26209914 (PMC12563882; doi:10.3390/ijms26209914)
Supplement: Supplementary file 1 [file ijms-26-09914-s001.zip › Supplementray Figures.pdf]

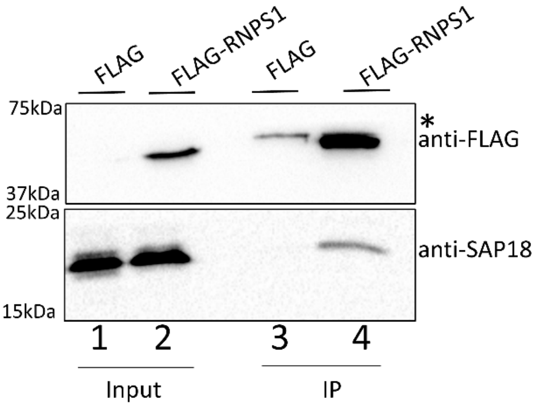

Supplementary Figure S1: RNPS1 interacts with SAP18: Plasmid expressing unfused FLAG (control) and FLAG-tagged RNPS1 is transiently transfected to HEK-293 cells. After 48 hours, cells were lysed and subjected to FLAG-IP. The eluate was subjected to Western blotting. Compared to unfused FLAG, FLAG-RNPS1 detected the interaction with SAP18 in IP. The asterisk (*) denotes the heavy chain of immunoglobulin in lane 3.

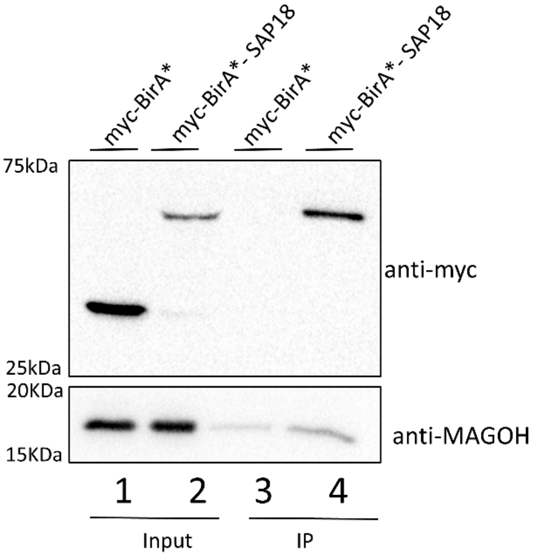

Supplementary Figure S2: Validation of BirA*-SAP18 interaction with MAGOH by streptavidin affinity purification: Stable Flp-In 293 cells expressing BirA*-SAP18 fusion proteins were supplemented with 50 µM biotin. Cells lysates were prepared and biotinylated proteins was recovered on streptavidin beads. Eluates obtained from streptavidin purification were analyzed by western blotting with antibodies against Myc (to visualize unfused BirA* and BirA*-SAP18) and MAGOH. Compared to BirA*, enrichment of MAGOH is detected in BirA*-SAP18 IP lane.
